# The rubber hand illusion evaluated using different stimulation modalities

**DOI:** 10.3389/fnins.2023.1237053

**Published:** 2023-09-14

**Authors:** Pamela Svensson, Nebojša Malešević, Ulrika Wijk, Anders Björkman, Christian Antfolk

**Affiliations:** ^1^Department of Biomedical Engineering, Faculty of Engineering, Lund University, Lund, Sweden; ^2^Department of Translational Medicine – Hand Surgery, Lund University and Skåne University Hospital, Malmö, Sweden; ^3^Department of Hand Surgery, Institute of Clinical Sciences, Sahlgrenska Academy, University of Gothenburg and Sahlgrenska University Hospital, Gothenburg, Sweden

**Keywords:** rubber hand illusion, stimulation modality, vibrotactile stimulation, electrotactile stimulation, mechanotactile stimulation

## Abstract

Tactile feedback plays a vital role in inducing ownership and improving motor control of prosthetic hands. However, commercially available prosthetic hands typically do not provide tactile feedback and because of that the prosthetic user must rely on visual input to adjust the grip. The classical rubber hand illusion (RHI) where a brush is stroking the rubber hand, and the user’s hidden hand synchronously can induce ownership of a rubber hand. In the classic RHI the stimulation is modality-matched, meaning that the stimulus on the real hand matches the stimulus on the rubber hand. The RHI has also been used in previous studies with a prosthetic hand as the “rubber hand,” suggesting that a hand prosthesis can be incorporated within the amputee’s body scheme. Interestingly, previous studies have shown that stimulation with a mismatched modality, where the rubber hand was brushed, and vibrations were felt on the hidden hand also induced the RHI. The aim of this study was to compare how well mechanotactile, vibrotactile, and electrotactile feedback induced the RHI in able-bodied participants and forearm amputees. 27 participants with intact hands and three transradial amputees took part in a modified RHI experiment. The rubber hand was stroked with a brush, and the participant’s hidden hand/residual limb received stimulation with either brush stroking, electricity, pressure, or vibration. The three latter stimulations were modality mismatched with regard to the brushstroke. Participants were tested for ten different combinations (stimulation blocks) where the stimulations were applied on the volar (glabrous skin), and dorsal (hairy skin) sides of the hand. Outcome was assessed using two standard tests (questionnaire and proprioceptive drift). All types of stimulation induced RHI but electrical and vibration stimulation induced a stronger RHI than pressure. After completing more stimulation blocks, the proprioceptive drift test showed that the difference between pre- and post-test was reduced. This indicates that the illusion was drifting toward the rubber hand further into the session.

## 1. Introduction

After a hand amputation, both motor and sensory functions are lost. The functionality and appearance of the lost hand can be restored to some extent by a prosthetic hand. However, many amputees show dissatisfaction with the functionality and lack of sensory feedback (Biddiss and Chau, 2007; Wijk and Carlsson, 2015). Sensory feedback is crucial for experiencing ownership of a body part or a prosthesis (Botvinick and Cohen, 1998; Ehrsson, 2004; Tsakiris and Haggard, 2005). Ownership of a prosthetic hand elicits a feeling that it is a part of the body and could also contribute to improved control of the prosthesis (Wijk and Carlsson, 2015). The sense of ownership is a key element, together with the sense of agency, which is a feeling that one controls the movement, to create prosthetic embodiment, where”the prosthesis is perceived as part of the body” (Zbinden et al., 2022). In addition, ownership is critical for prosthetics acceptance (Graczyk et al., 2019) and amputees with high levels of prosthesis ownership have significantly lower levels of phantom limb pain (PLP) and residual limb pain (RLP) (Bekrater-Bodmann et al., 2021).

The Rubber Hand Illusion (RHI) is a well-established model for studying body ownership. The classical RHI was described by Botvinick and Cohen (1998), where brushing both a rubber hand and the participant’s own hand synchronously for 10–110 s induced a sense of ownership of the rubber hand (Ehrsson et al., 2005; Kammers et al., 2009). Brushing both the rubber hand and the participant’s hidden hand provides a visuotactile, modality-matched stimulation. On the other hand, brushing the participant’s own hand asynchronously, where the delay is longer than 300 ms (Shimada et al., 2009), related to the brushing on the rubber hand, does not induce the RHI (Kammers et al., 2009).

Ehrsson (2004) used functional magnetic resonance imaging (fMRI) to assess brain activity in healthy subjects during the RHI. They found that activity in the premotor and intraparietal cortex was correlated to the subjective measure of vividness, which defined how realistic and life-like the participants perceived the RHI. Furthermore, Ehrsson et al. (2008) assessed the RHI in eighteen unilateral transradial amputees showing that the RHI was enhanced when brushing the index finger of the rubber hand and the referred phantom index finger on the residual limb compared to the contralateral arm area. Interestingly, Rosén et al. (2009) showed that the artificial rubber hand does not have to imitate the appearance of a biological hand to be perceived as belonging to the own body. They performed the RHI on five upper limb amputees who experienced referred sensations on the same phantom finger when brushing a finger of the robotic-like hand and on the site of the referred area of sensation. The classical RHI is based on visuotactile stimulation on stationary parts. The effects of both passive and active movements on the RHI have been evaluated (Kalckert and Ehrsson, 2014) showing that the RHI was equally strong in both active and passive movement, suggesting that any combination of multisensory stimulation can induce the RHI (Kalckert and Ehrsson, 2014).

Human skin comprises hairy skin and hairless skin (glabrous skin, mainly found on the palms and sole of the feet). Glabrous skin is innervated by four types of mechanoreceptors which respond to different aspects of touch (Johansson and Vallbo, 1979; Purves et al., 2018). Yet another aspect of touch is mediated by C-tactile (CT) afferents, found in hairy skin, that respond to soft brush stroking with velocities at 1–10 cm/s (Löken et al., 2011) and code for pleasant touch (Vallbo et al., 1999). Prior studies have shown that a light touch with a slow stroking speed was deemed more pleasant and provided higher levels of subjective ownership and embodiment during the RHI than a fast-stroking speed (Crucianelli et al., 2013; Lloyd et al., 2013). Furthermore, stimulating the hairy skin of the hand, rather than the glabrous skin, showed a greater proprioceptive drift (Lloyd et al., 2013).

Vibrotactile, electrotactile, and mechanotactile feedback are commonly used when investigating non-invasive sensory feedback in hand prosthetics. If these modalities are used in solitary there can be a mismatch between what is seen and what is felt. E.g., if a person sees a force being acted on a hand prosthesis but the stimulation felt is vibration, this can be seen as a visuotactile mismatch.

D’Alonzo and Cipriani (2012) showed that synchronous tapping induced a vivid RHI but less than during synchronous brush stroking. Furthermore, vibrotactile stimulation induced ownership of the rubber hand during mismatched modality stimulation, but the RHI was more vivid during brush-vibration than tapping-vibration. D’Alonzo et al. suggested that brush-vibration induce a more vivid RHI because both types of stimulation causes activation of the same mechanoreceptors, which is not the case during tapping-vibration. Shehata et al. (2020) compared the classical RHI (brush-brush) to a modified RHI using mechanotactile feedback (tapping-tapping). The mechanotactile stimulation was also evaluated together with motor control of a simulated prosthesis. They showed that tapping-tapping evoked similar embodiment responses as brush-brush. Furthermore, controlling a prosthesis (grasping objects) showed high agency responses, which was not influenced when adding mechanotactile sensory feedback. However, with asynchronous sensory feedback, while grasping objects with the prosthesis, the agency responses were lower than without sensory feedback. Schütz-Bosbach et al. (2009) investigated roughness perception where participants watched a rubber hand being stroked by soft or rough fabric while they received synchronous or asynchronous tactile stimulation that was either congruent or incongruent with respect to the sensory quality of the material touching the rubber hand. They showed that there was no effect of roughness on the rubber hand illusion. Mulvey et al. (2009) used non-invasive transcutaneous electrical nerve stimulation (TENS) and modified version of the rubber hand illusion. It was shown that TENS paraesthesiae can be made to feel like it is emanating from a prosthetic and in healthy participants. D’Alonzo et al. (2019) assessed the importance of virtualization of sight and touch on artificial embodiment. They showed that virtualization decreased embodiment. Pinardi et al. (2020) used vibrotactile stimulation in the form of the tendon vibration illusion and a modified version of the rubber hand illusion (the moving hand illusion) in their experiments. They found that ownership and agency are independently processed, and presence of the efferent component modulates sensory feedbacks salience. Page et al. (2018) used the rubber hand illusion to assess the embodiment of a 3-D printed prosthetic hand endowed with force sensors using microelectrode arrays implanted in the residual median and ulnar nerve of an amputee. The participant experienced embodiment of the prosthesis in several experimental conditions.

The aim of this study was to assess how well mechanotactile, vibrotactile and electrotactile feedback can induce the RHI in able-bodied volunteers and transradial amputees. We hypothesize that stimuli that provide a sensation similar to brush stroking would provide a stronger RHI. Such sensations can be elicited by electrotactile and vibrotactile stimulation since the sensations could match the brush stroking spatially, meaning that the stimulus can be felt on a larger area on the finger. Additionally, these stimulations could give a tingling sensation which might be similar to brushstrokes. On the other hand, stimulation with pressure would hypothetically only stimulate a smaller area, resulting in a weaker RHI than the other stimulation types.

## 2. Methods

### 2.1. Participants

This study includes two cohorts of participants; twenty-seven able-bodied, right-handed individuals (18 males and 7 females; median age, 34 years; range 25–60 years) and three unilateral transradial amputees (3 males, aged 22, 42, and 47 years). In this study, the amputees are referred to as A1, A2, and A3. A1 was amputated on the right side 1 year prior to the experiment. He displayed a map of referred sensations or phantom hand map (PHM) (Björkman et al., 2016) on the distal part of the residual forearm. On this PHM, he experienced referred sensations from the volar side of the thumb, index and little fingers, as well as part of the palm. The PHM was defined by using a pen as previously described (Ehrsson et al., 2008; Rosén et al., 2009; Björkman et al., 2016). A2 was amputated on the left side 32 years ago. He experienced a shortened phantom limb, a phenomenon called telescoping, with the whole phantom hand intact and he also perceived that he could move the phantom fingers. However, he did not experience any referred sensations or PHM. A3 was amputated on the right side 18 years ago. He could perceive the movement of the phantom thumb and little finger when activating the residual forearm muscles but experienced no referred sensations or PHM. All participants used a myoelectric prosthesis, and A3 had the prosthesis attached with osseointegration.

The study was approved by the Swedish Ethical Review Authority (DNR 2021–03630) and was conducted in accordance with the tenets of the Declaration of Helsinki. All participants were informed about the contents of the experiments, both verbally and in writing, and gave their informed and written consent.

### 2.2. Equipment

**Figure 1:**
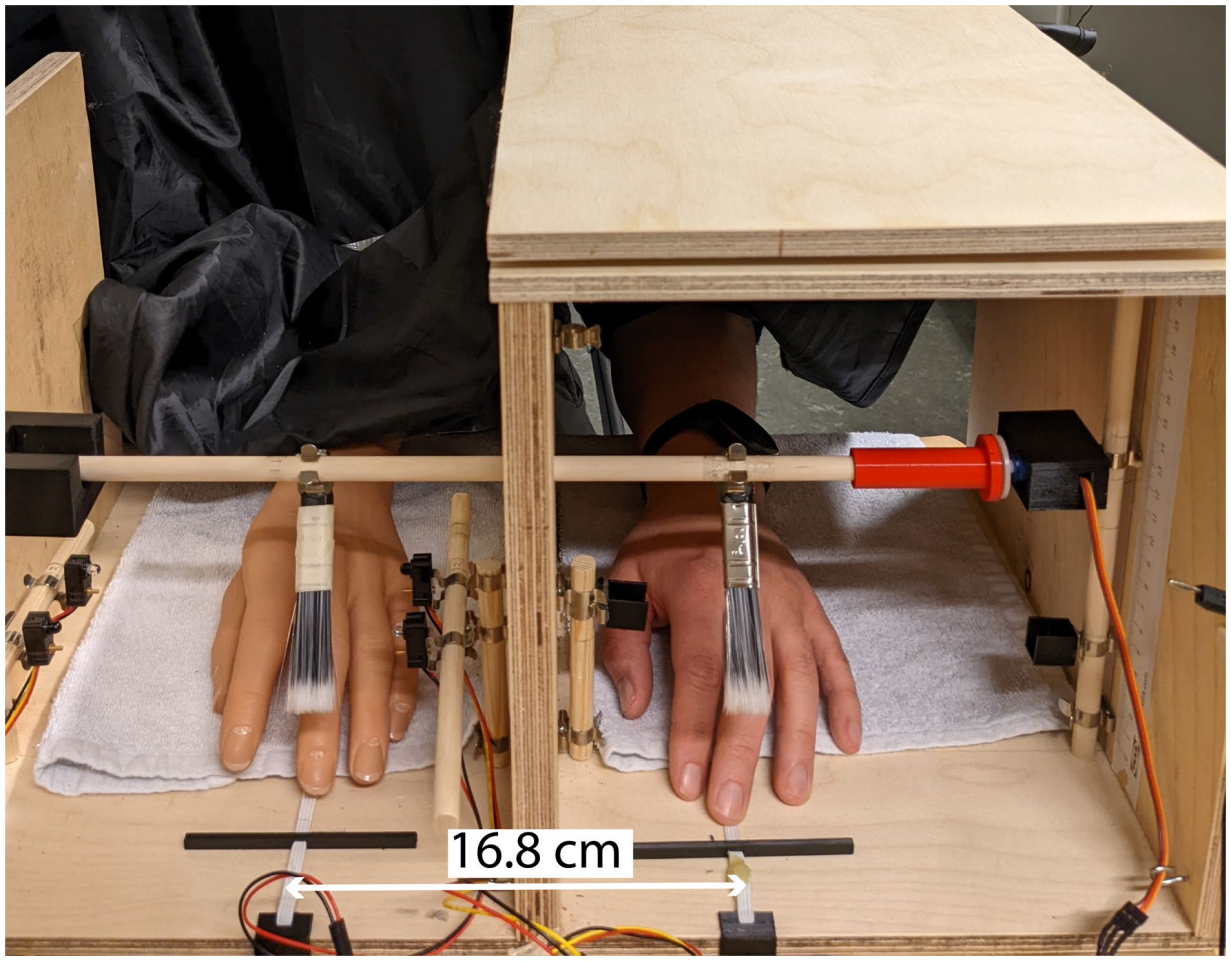
The experimental setup, where brush stroking was applied to the rubber hand and the participant’s biological hand (classic Rubber Hand Illusion). The white rubber band was used as a guide for where to position the long finger. The distance between the hands was 16.8 cm.

The setup for the RHI included a box (RHI box) with openable lids to obscure and reveal the rubber hand during the experiment (see Figure 1). The RHI box was divided into two compartments, where a life-like rubber hand was placed in an anatomically congruent position on one side, and the participant’s hand was placed on the other side in the same posture as the rubber hand. The RHI box contained customized racks to adjust the position of sensors and actuators. One continuous rotation servo, FS90R (Feetech RC Model Co., Ltd., Shenzhen, China), was used to rotate a rod onto which brushes were attached. Two pairs of infrared (IR) sensors (Adafruit Industries LLC, NYC, United States) were used to detect the onset and cessation of the brush stroking. The IR sensors were used to keep count of the brushstrokes and to send an on and off signal to the actuators, which provided stimulation to the hidden biological hand. Three different devices were used to provide different tactile stimulations on the participant’s hidden long finger and the distal part of the residual forearm in the amputees. An HS-40 Nano analogue servo motor (HI-TEC RCD, USA) provided mechanotactile feedback on the hidden biological hand (similar to what was done in Wijk et al. (2019)). The motor was attached to a rod placed above the hidden biological hand, and the height was adjusted to provide a light touch on the skin. For vibrotactile feedback, an eccentric rotating mass motor (ERM) (Vibrating Mini Motor Disc ID 1201, 11,000 RPM, Adafruit Industries LLC, NY, United States) was used and secured with tape to be kept in place during the experiment. For the electrotactile stimulation, an electrical stimulator was used to produce biphasic charge-balanced cathodic-first current-controlled pulses. The amplitudes ranged from 0.1 mA to 10 mA with a resolution of 0.1 mA, and the stimulation frequency was set to 100 Hz. The electrical stimulation was delivered through self-adhesive Pals electrodes (Axelgaard Manufacturing Co., Lystrup, Denmark).

### 2.3. Experimental setup and protocol

In this study, we investigated four types of feedback (brushstrokes, pressure/force, vibrations, and electrical stimulation), which were tested on different parts of the hand (hairy skin and glabrous skin), with and without time delay (synchronous and asynchronous). The given stimulus was applied to the participant’s hidden biological hand while the rubber hand always received brushstrokes as the stimulus. However, to limit the number of combinations, asynchronous stimulation was only performed when the brush was stroking both the rubber hand and the biological hand. Testing these conditions gave a total of 10 combination blocks. Throughout the paper, the stimulation blocks are coded as [Timing] [Hidden hand stimuli]–[Skin type] (Table 1). For example, SBG would denote that stimuli on both the hidden hand and the rubber hand were synchronous (without time delay) and a brush was used to stimulate the hidden hand (a brush was always used to stimulate the rubber hand) and that stimuli on both the hidden hand and rubber hand was applied to what would be conceived as glabrous skin. Each participant took part in one session with ten stimulation blocks, where each block provided 30 stimulations, lasting approximately 100 s. The ten stimulation blocks were randomized within the session and among the participants. The participant had a 1–2 min break between each block and was instructed to stand up, relax and walk around during the break. The participants disengaged completely from the experimental setup during the break. The experimental setup and protocol were the same for able-bodied participants and amputees, if not stated otherwise.

**Table 1:** Abbreviation for the coding of the 10 stimulation blocks.

Timing	Hidden hand stimuli	Skin type
Asynchronous (A)	Brush (B)	Hairy skin (H)
Synchronous (S)	Pressure (P)	Glabrous (G)
	Vibration (V)	
	Electrical (E)	

#### 2.3.1. Identification of stimulation amplitude

The initial step of the experimental protocol was to set the amplitude for the electrical stimulation for the participant. The anodal electrode (rectangular 7 × 10 cm Pals electrode) was placed on the ventral side of the forearm. The cathode was placed on the proximal phalanx of the long finger in the case of the able-bodied participants and on the distal part of the residual forearm in the amputees in the case of amputees (circular 2.5 cm in diameter Pals electrode) and secured with tape to maintain stable contact skin throughout the experiment. A two-second stimulation with a frequency of 100 Hz and in increments of 0.2 mA was given to define; (a) sensory threshold, (b) pain/uncomfortable stimulation, and (c) the level where the participant felt the stimulation distinctly (stimulation level). Identifying the thresholds was done on glabrous and hairy skin separately. The mean stimulation level on glabrous and hairy skin for participants with intact hands was 1.67 mA and 1.98 mA. The stimulation level for the amputees was set by increasing the intensity until they felt a distinct stimulation. The results for the identification of stimulation amplitude can be seen in Supplementary Figure S1.

#### 2.3.2. Rubber hand illusion experimental session

The participants were seated comfortably in a chair in front of a table facing the experimenter. The chair was set at a proper height, and the armrest was positioned, so the hidden biological hand attained a relaxed position in the RHI box. All jewelry and watches were removed to make the biological hand visually similar to the rubber hand. The participants wore noise-cancelling headphones and listened to white noise during the experiments to remove any auditory cues. A sheet covered the participant’s shoulder to obscure the arm being studied.

**Figure 2:**
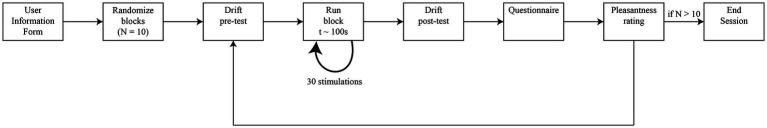
Experimental protocol for one session.

The participant’s left hand was positioned in the RHI box at a fixed distance (16.8 cm) in relation to the rubber hand. For each of the ten blocks, the following sequence of actions were performed. After completing the setup (depending on the hidden stimuli type) the following steps were performed: (1) the lid of the box was closed, and the participant was asked to perform the proprioceptive drift pre-test, (2) the experimenter opened the lid, and the participant was asked to focus on the rubber hand, (3) the stimulation paradigm was administered (30 stimulations were provided), (4) when a stimulation block was finished, the experimenter closed the lid, and the participant was asked to perform the proprioceptive drift post-test, (4) the participant continued with the questionnaire (9 questions) and finished with (5) rating the pleasantness of the stimulus. The different parts of the experiment can be seen in Figure 2. After completing one stimulation block and the RHI tests, the participants took a break while the experimenter prepared for the next block.

**Figure 3:**
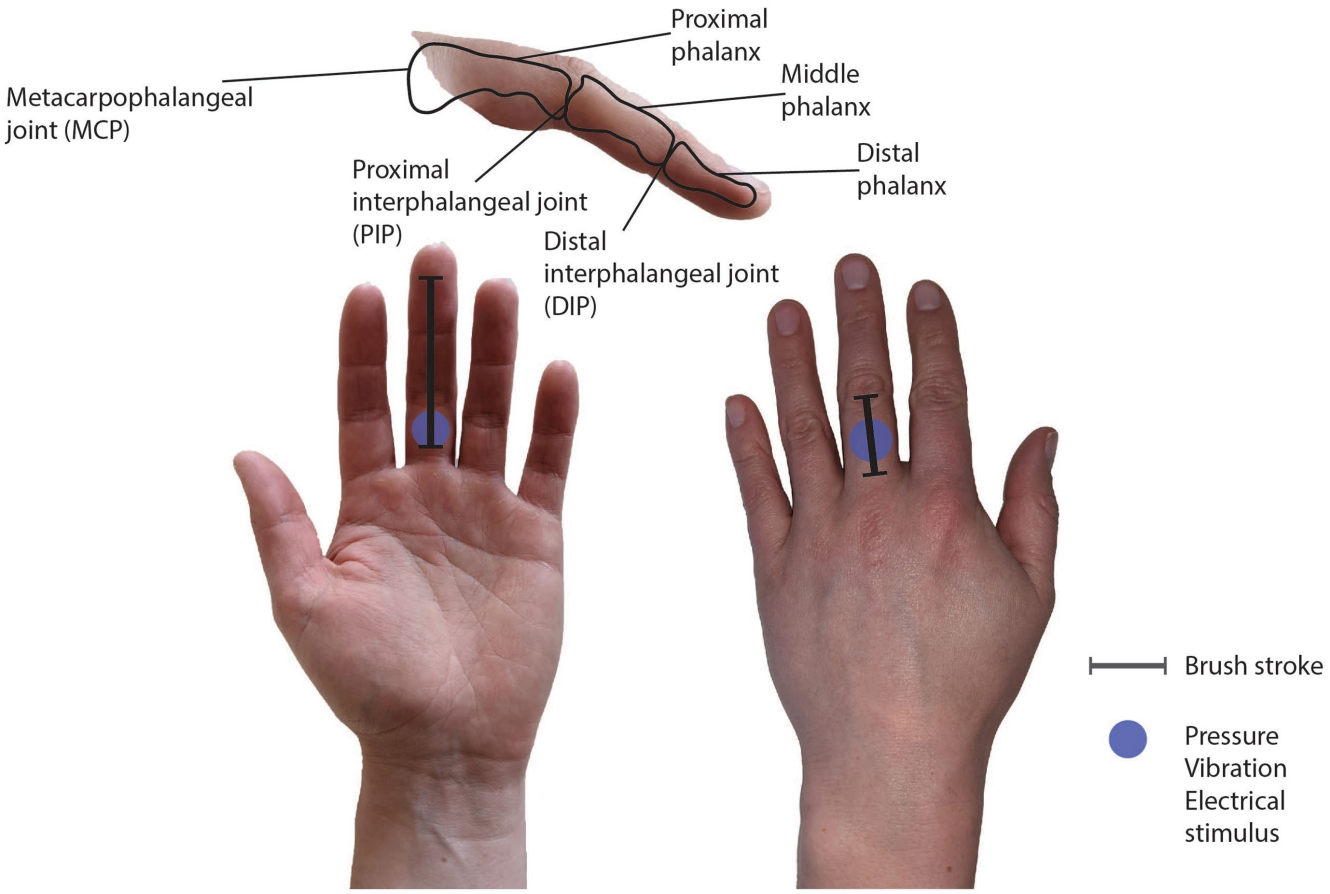
Bones and joints of the finger (upper picture). (Lower pictures) The lines on the long finger show the brush stroke path, and the blue dot shows the position of the pressure, vibration, and electrical stimulation. On the glabrous skin (left), the brush stroking started on the proximal phalanx and ended on the distal phalanx. On the hairy skin (right), the brush stroking started on the proximal phalanx and ended at the MCP joint.

The SBH condition follows the original experiment done by Botvinick and Cohen (1998). The rubber hand was positioned in a congruent position to the hidden, biological hand, and the participant was instructed to relax the fingers of the hidden hand in a position similar to the rubber hand where the fingers were slightly bent. The brush stroking started at the proximal phalanx of the participant’s long finger and the rubber hand and ended on the long finger’s metacarpophalangeal joint (MCP) joint. For the SBG condition, the fingers of both the rubber hand and the biological hand were fully extended to match the brush path, which started at the proximal phalanx and ended at the distal phalanx. For the asynchronous condition (ABH and ABG), the brush stroked the hidden biological finger approximately 500 ms after the brush stroking on the rubber hand. During mechanotactile, vibrotactile, and electrotactile feedback, the actuators were placed on the proximal phalanx of the long finger (see Figure 3). This position was chosen because brushing on the rubber hand started on the proximal phalanx.

The stimulation was applied differently to the amputees compared to the able-bodied participants. The amputees without a phantom hand map (A2 and A3) received stimulation on the distal part of the residual limb at a central point, in conjunction with brushstrokes given on the dorsal and volar sides of the rubber hand. As for A1, who had a PHM on the residual limb, the stimulation was applied on the phantom little finger during brushstrokes on the little finger of the rubber hand. The phantom little finger was chosen for a practical reason: distancing from scars where the sensitivity was reduced. On all three amputees the brush stroking and the mechanotactile stimulation were applied manually by the experimenter to the amputees’ residual limbs due to mechanical constraints of the experimental setup. Furthermore, A3 was omitted from the condition using electrotactile stimulation as equipment used did not allow for a high enough stimulation current.

### 2.4. Outcome measures

Three tests were performed for each stimulation block to assess the RHI; proprioception drift pre- and post-test, questionnaire, and pleasantness test. After completing all stimulation blocks, the participant could by choice, convey comments about the RHI experience.

#### 2.4.1. Proprioceptive drift test

The proprioceptive drift test is a pointing test that was done before (drift pre-test) and after (drift post-test) each stimulation block according to Tsakiris and Haggard (2005). The proprioceptive drift was calculated as the difference between the pre- and post-test (Tsakiris and Haggard, 2005). A positive drift indicated a drift towards the rubber hand, and a negative drift indicated a drift away from the rubber hand.

#### 2.4.2. Questionnaire

Directly after the proprioceptive drift post-test, the participant filled out a questionnaire containing nine questions adapted from Botvinick and Cohen (1998) (see list in Supplementary materials). The participant rated the statements using a seven-point visual-analogue scale (VAS). This scale ranged from −3 (“absolutely certain that it did not apply”), 0 (“uncertain whether it applied or not”), and + 3 (“absolutely certain that it did apply”). Three statements assessed the illusion, which referred to if the sensation was felt on the rubber hand and if the rubber hand was felt as if it were one’s hand. Statement S1 and S2 assessed experience of referred touch, and S3 assessed the ownership of a rubber hand. The other six statements served as controls for suggestibility and task compliance. The statements were randomized after each block and for each participant.

In order to assess how many participants experienced the RHI, an ownership criterion was used (Zbinden and Ortiz-Catalan, 2021): Participants who scored higher than the neutral rating, 0, for the mean score of the illusion statements during synchronous brush stroking condition and had one point or more than the asynchronous brush stroking condition in VAS. The criterion was used on all data, including both hairy and glabrous skin, but the criterion was also employed on the skin types separately. It is unknown why some persons are susceptible to RHI and others are not (Riemer et al., 2019). The classification of non-responders and responders is often based on subjective self-reports such as the RHI questionnaire and not according to objective measures. Statistical analyses were initially carried out to determine whether the number of non-responders were significantly different in hairy and glabrous skin. In the event that no such significant difference was observed, subsequent analyses were conducted using the consolidated data comprising both glabrous and hairy skin.

The mean rating of illusion statements was compared between asynchronous and synchronous brush stroking to test whether the RHI was induced. Thereafter the mean rating of illusion statements was compared to the mean rating of control statements for all the stimulus types. The mean rating of illusion statements should have a higher rating than the control statements if the participants experienced an RHI (Botvinick and Cohen, 1998; Ehrsson et al., 2008; Rosén et al., 2009). Tests were performed on hairy and glabrous skin separately to assess differences in the mean rating of illusion and control statements between the two skin types. The results from the different stimuli were compared in order to assess if a specific type of stimuli induced a more vivid RHI.

#### 2.4.3. Pleasantness rating

After the questionnaire, the participants were asked to rate the pleasantness of the stimulation on a VAS which is a subjective measure (Löken et al., 2009). The scale ranged from −3 (“unpleasant”), 0 (“indifferent”) to +3 (“pleasant”). The rating was done by moving the indicator on the scale with a computer mouse using the contralateral hand.

### 2.5. Statistical analysis

Statistical analyses were performed using Linear Mixed Models for the outcome measures proprioceptive and pleasantness with factor skin type (glabrous, hairy) and stimulation type (brush asynchronous, brush synchronous, vibrotactile, electrotactile and mechanotactile). For the outcome measure questionnaire, a Linear Mixed Model with factors skin type (glabrous, hairy), stimulation type (brush asynchronous, brush synchronous, vibrotactile, electrotactile and mechanotactile) and question type (control, illusion) was used. Normality of residuals were checked visually using Q-Q plot. In all cases, the residuals were deemed to be normally distributed. Post-hoc tests were performed using Tukey’s multiple comparisons method. All reported *p*-values are adjusted for multiplicity and *p* < 0.05 is considered significant. All calculations were performed using GraphPad Prism (version 10.0.2, GraphPad Software, Boston, MA, United States).

## 3. Results

### 3.1. Proprioceptive drift

#### 3.1.1. Able-bodied participants

There was a higher drift towards the rubber hand following synchronous brush stroking (2.99 ± 3.35 cm) compared to asynchronous brush stroking (1.17 ± 3.16 cm).

**Figure 4:**
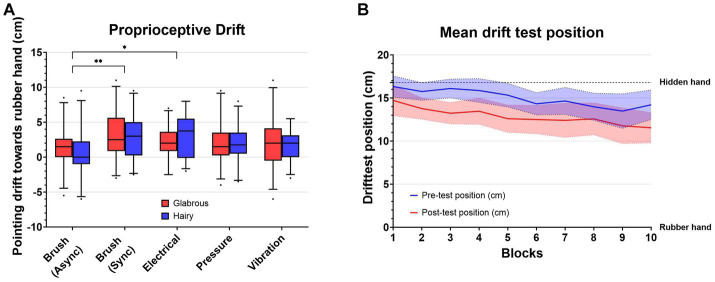
**(A)** Proprioceptive drift for each stimulus: Brush asynchronous stroking (BA), brush synchronous stroking (BS), pressure (P), vibration (V), and electrical stimulation (E). The proprioceptive drift towards the rubber hand was significantly greater with synchronous brush stroking than asynchronous brush stroking (*p* < 0.001) (^*^ ≡ *p* < 0.05, ^**^ ≡ *p* < 0.01, ^***^ ≡ *p* < 0.001) **(B)**. **(B)** Mean values from the drift test (cm) with a 95% CI for all stimulus types. Panel **(B)** shows that the pointing position already drifted towards the rubber hand before starting each block. 0 cm indicates the position of the rubber hand, and 16.8 cm is the position of the hidden hand.

When comparing proprioceptive drift as a result of the different stimuli, the highest proprioceptive drift was seen with brush stroking, followed by electrical stimulation (2.65 ± 2.68 cm), vibration (2.00 ± 2.84 cm), and pressure (1.93 ± 2.83 cm) (Figure 4A). A Linear Mixed Effect model was used to analyze the effect of stimulation type and skin type on proprioceptive drift. The results showed that there was not a statistically significant interaction effect of skin type and stimulation type (*F* (4, 100) = 1.480, *p* = 0.2140). Simple main effects analysis showed that skin type did not have a statistical significant effect on proprioceptive drift (*F* (1, 25) = 0.2030, *p* = 0.6562). Simple main effects analysis showed that stimulation type did have a statistically significant effect on proprioceptive drift (*F* (4, 100) = 4.428, *p* = 0.0025). Post-hoc tests showed a significant difference between Brush (asynchronous) and Brush (Synchronous), *p* = 0.0021, and between Brush (asynchronous) and Electrotactile stimulation, *p* = 0.0143.

Both the pre-test and the post-test values tended to drift toward the position of the rubber hand after the more stimulations were completed (Figure 4B). A paired t-test was also used to analyse the difference in pointing positions for the first and last stimulation blocks. The pointing position was significantly closer to the rubber hand in the last block both in the pre-test [*t* (52) = 2.43, *p* = 0.02] and in the post-test [*t* (52) = 3.33, *p* = 0.003].

#### 3.1.2. Amputees

**Figure 5:**
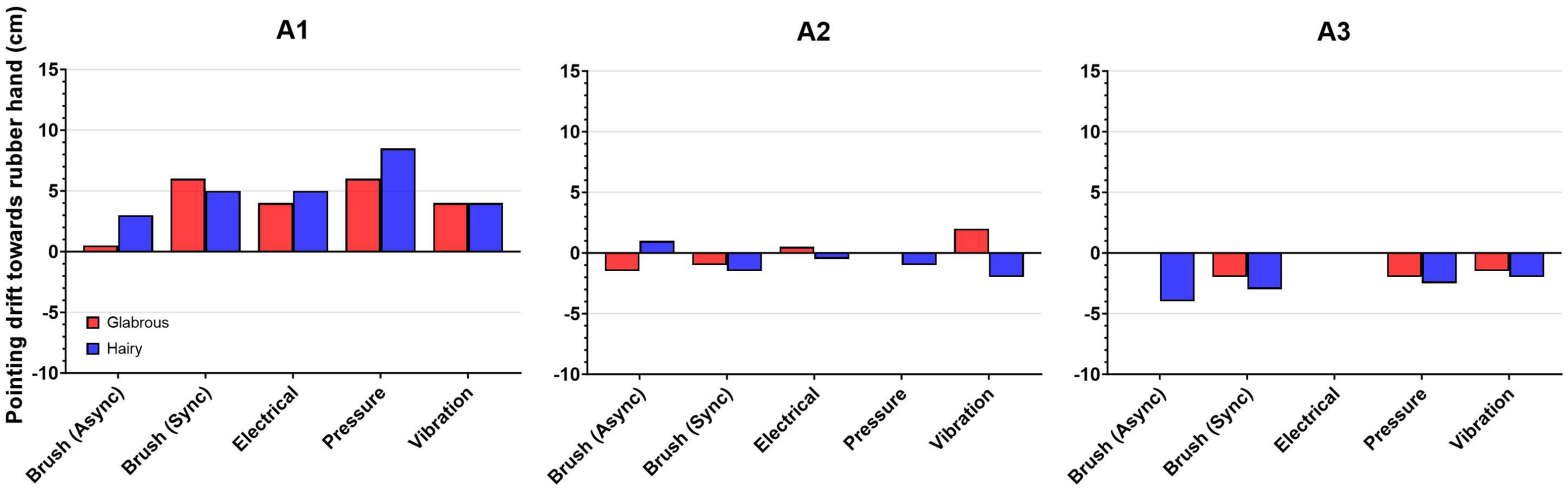
Proprioceptive drift for each amputee: The amputee who had a more recent amputation A1, time (since amputation 1 year) had greater drift towards the rubber hand than the other two amputees A2, time since amputation 32 years and A3, time (since amputation 18 years).

A1 had the highest drift towards the rubber hand when pressure was applied on the forearm, followed by brush stroking on the dorsal side of the rubber hand (Figure 5). For amputees A2 and A3, the drifts were less pronounced than in the case of A1 but present in most of the stimulation types.

### 3.2. Questionnaire

#### 3.2.1. Able-bodied participants

**Figure 6:**
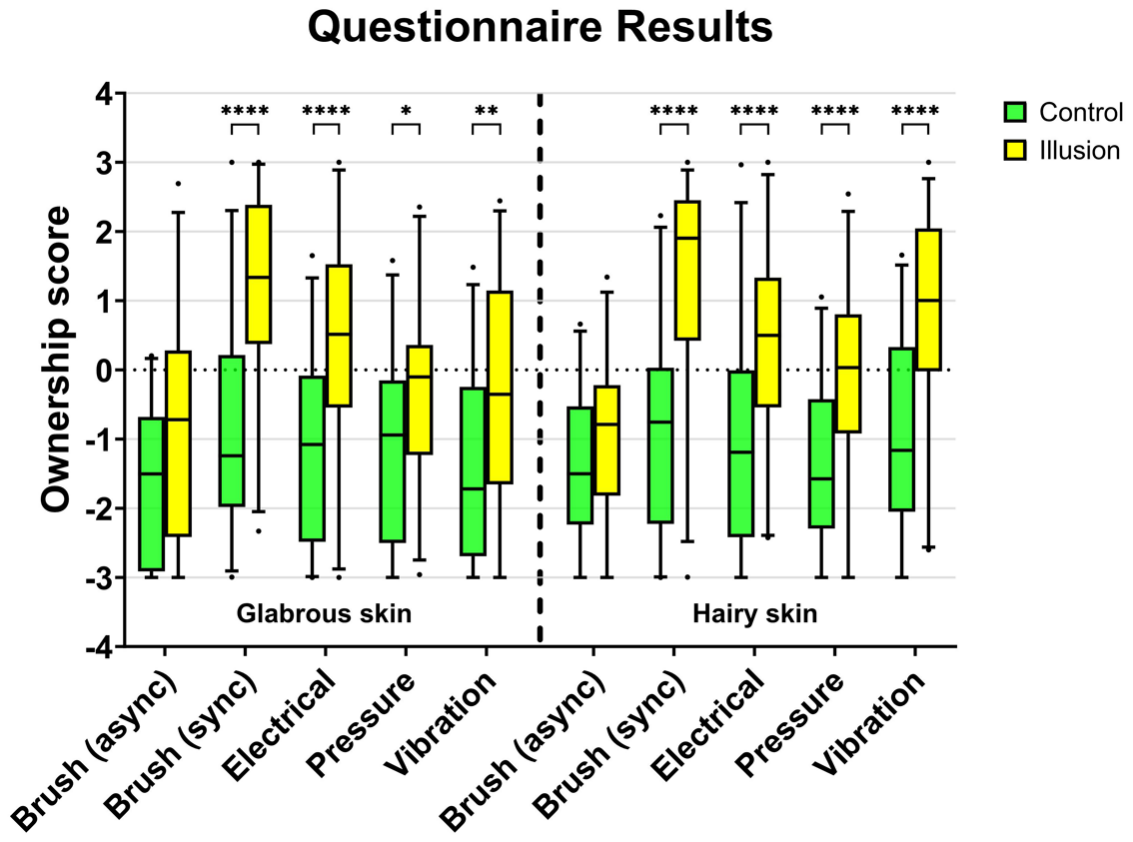
Results of the questionnaires. The three illusion statements are marked with (*), and the rest (S4–S9) are control statements. The results show all stimulus types. The traditional RHI had a significantly higher rating than other stimuli. However, all types of stimuli induced the illusion.

The illusion statements (S1-S3) had a higher, more positive, rating than the control statements (S4-S9) (1.26 and − 0.88) during synchronous brush stroking (Figure 6). In contrast, the rating was −0.91 (illusion statements) and − 1.50 (control statements) during asynchronous brush stroking.

In the current study 6 of 27 (22.2%) were non-responders following stimulation on hairy skin and 9 of 27 (33.3%) were non-responders following stimulation of glabrous skin. However, the number of non-responders was not significantly different in hairy and glabrous skin. Therefore, the final tests were made on all data, including results from stimulation on hairy and glabrous skin.

A Linear Mixed Effect model was used to analyze the effect of stimulation type, skin type and question type on the ownership score as defined by the questionnaire. The results showed that there was a statistically significant interaction effect of skin type and stimulation type (*F* (4, 100) = 3.169, *p* = 0.0170), of stimulation type and question type (*F* (4, 100) = 17.35, *p* < 0.0001) and of stimulation type and skin type and question type (*F* (4, 100) = 2.618, *p* = 0.0394). There was no statistically significant interaction effect of skin type and question type (*F* (1, 25) = 1.174, *p* = 0.2889). Simple main effects analysis showed that skin type did not have a statistically significant effect on the ownership score (*F* (1, 25) = 2.788, *p* = 0.1074). Simple main effects analysis showed that stimulation type did have a statistically significant effect on ownership score (*F* (4, 100) = 18.63, *p* < 0.0001). Simple main effects analysis showed that question type did have a statistically significant effect on ownership score (*F* (1, 25) = 57.85, *p* < 0.001).

Post-hoc tests were carried out for glabrous skin type in the control vs. illusion condition and the results showed no statistically significant difference between control and illusion scores for the Brush (Asynchronous) condition (*p* = 0.2245). The results also showed a statistically significant difference between control and illusion scores for the Brush (Synchronous) condition (*p* < 0.0001), the Electrotactile condition (*p* < 0.0001), the Mechanotactile condition (*p* < 0.0394) and the Vibrotactile condition (*p* < 0.0046). Similarly, tests were performed for the hairy skin type with similar outcomes. The results showed no statistically significant difference between control and illusion scores for the Brush (Asynchronous) condition (*p* = 0.9524). The results also showed a statistically significant difference between control and illusion scores for the Brush (Synchronous) condition (*p* < 0.0001), the Electrotactile condition (*p* < 0.0001), the Mechanotactile condition (*p* < 0.0001) and the Vibrotactile condition (*p* < 0.0001).

#### 3.2.2. Amputees

**Figure 7:**
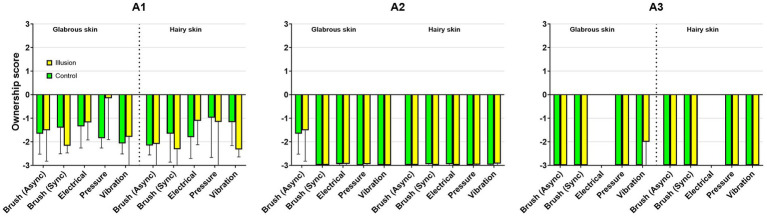
The bar plot shows the results of the questionnaire for each amputee. The two amputees who had lost their hand many years ago A2, time since amputation 32 years and A3, time (since amputation 18 years) showed an apparent disagreement to the statements in the questionnaire.

The amputees disagreed with the illusion statements (Figure 7). A1 rated one of the illusion statements, “It seemed as though the touch I felt was caused by the brush touching the rubber hand” at 1.04, similar to that of one of the control statements, “It seemed as if the touch I was feeling came from somewhere between my own hand and the rubber hand.”

### 3.3. Pleasantness ratings

#### 3.3.1. Able-bodied participants

**Figure 8:**
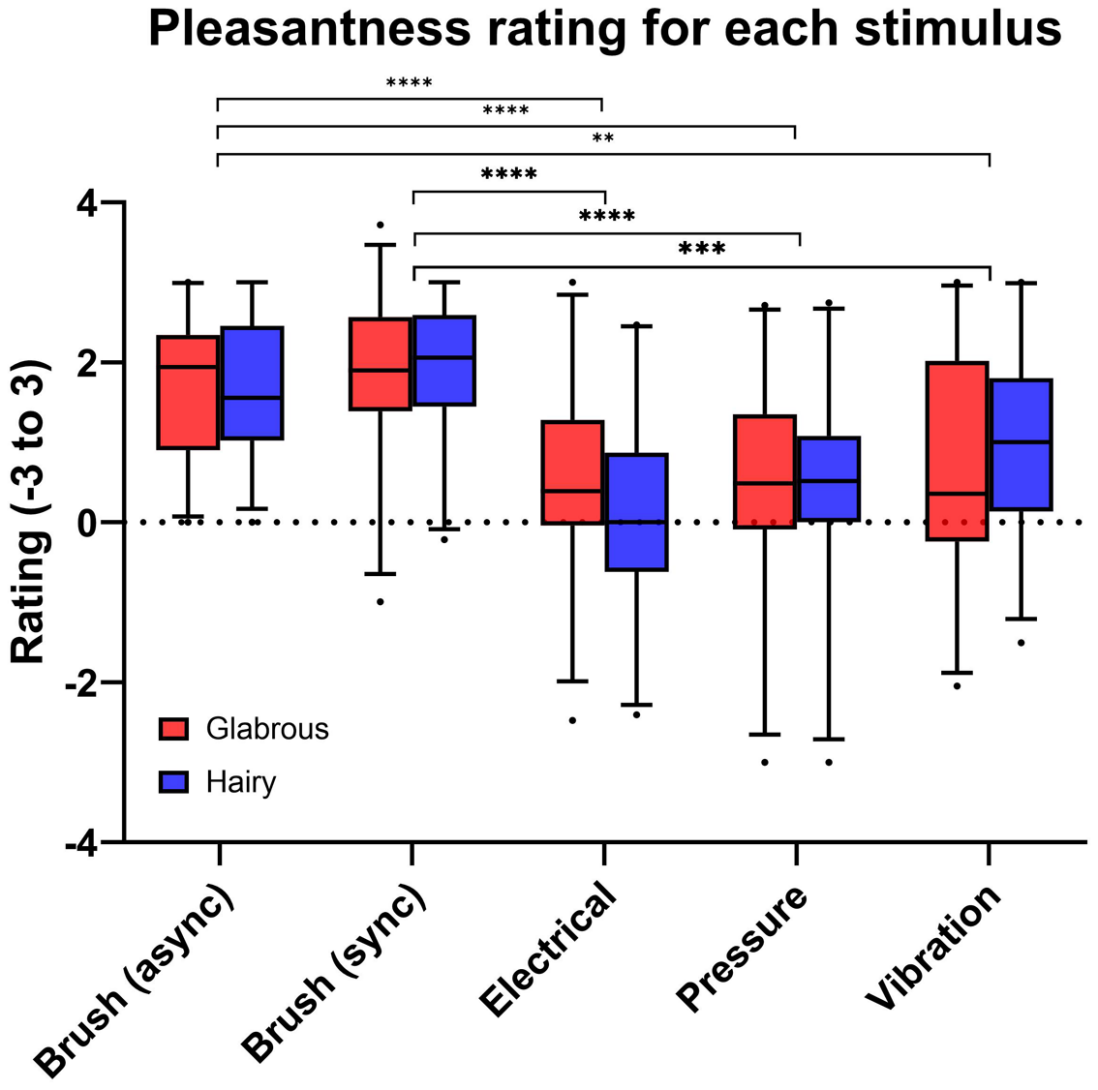
Pleasantness rating for each type of stimulus, where brush stroking was experienced as the most pleasant (^*^ ≡ p < 0.05, ^**^ ≡ *p* < 0.01, ^***^ ≡ *p* < 0.001).

A Linear Mixed Effect model was used to analyze the effect of stimulation type and skin type on the pleasantness rating. The results showed that there was not a statistically significant interaction effect of skin type and stimulation type (*F* (4, 95) = 2.241, *p* = 0.0703). Simple main effects analysis showed that skin type did not have a statistically significant effect on pleasantness (*F* (1, 25) = 0.002822, *p* = 0.9581). Simple main effects analysis showed that stimulation type did have a statistically significant effect on pleasantness (*F* (4, 100) = 17.74, p < 0.0001). Post-hoc tests showed a significant difference between Brush (asynchronous) and Electrotactile stimulation (*p* < 0.0001), Mechanotactile stimulation (*p* < 0.0001) and Vibrotactile stimulation (*p* = 0.0032). The post-hoc tests also showed a statistically significant difference between Brush (synchronous) and Electrotactile stimulation (*p* < 0.0001), Mechanotactile stimulation (*p* < 0.0001) and Vibrotactile stimulation (*p* = 0.0003) (Figure 8).

#### 3.3.2. Amputees

**Figure 9:**
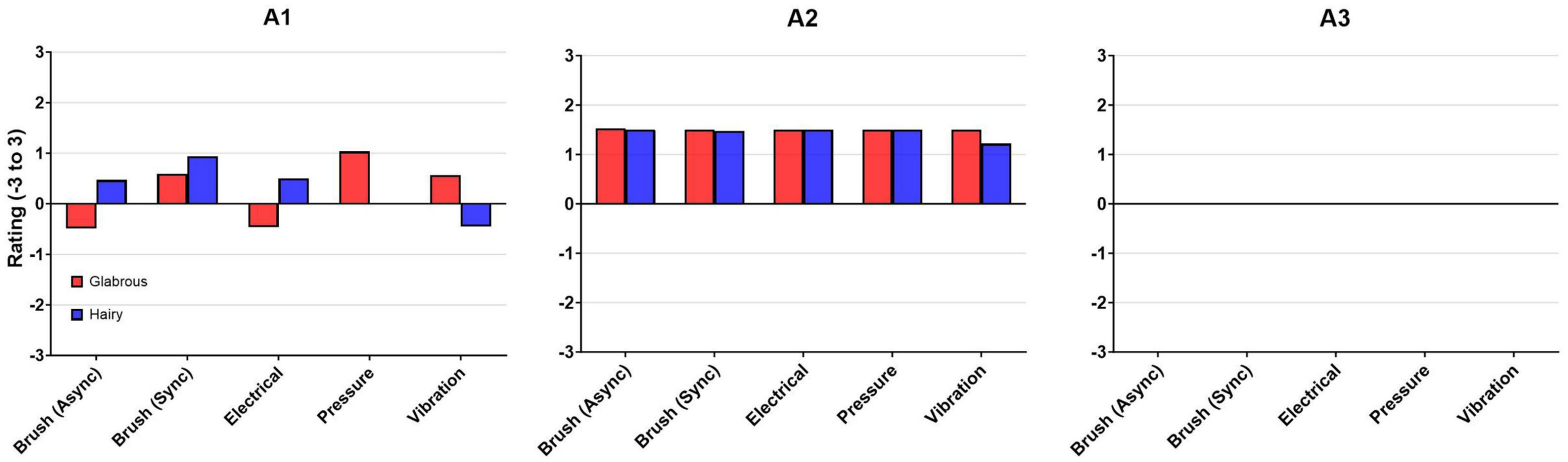
Pleasantness rating for each stimulus: Brush (B), pressure (P), vibration (V), and electrical stimulation (E).

The pleasantness rating for each stimulus can be seen in Figure 9. A1 mentioned that pressure was most pleasant (rating: 1.03) and was rated higher than the brush (rating: 0.94) on glabrous skin. A2 rated all the stimulation types similarly pleasant, 1.20–1.56. A3 experienced all the stimulation modalities as either pleasant or unpleasant (neutral pleasantness ranking).

## 4. Discussion

Previous studies have shown that if a hand amputee perceives their prosthetic hand as their own, they tend to use it more intuitively (Wijk et al., 2020). In order for this to happen, sensory feedback from the prosthesis is essential. In research, mechanotactile, vibrotactile, and electrotactile stimulation are common non-invasive methods to convey sensory feedback from a hand prosthesis to the amputee. This study assesses to what extent different types of stimuli, both modality-matched and mismatched can induce the RHI. Furthermore, it also assesses if the possibility of inducing the RHI is different depending on if the stimulus is applied to the glabrous or hairy skin in the hand.

Of the typical sensory feedback modalities employed in prosthetic hands research, and by extension potentially usable in general human machine interfaces, vibrotactile was perceived as the most pleasant. Electrotactile feedback provided for the greatest drift toward the rubber hand in the proprioceptive drift test. Electrotactile and vibrotactile also showed the best results in the questionnaires. Vibrotactile feedback devices are typically quite uncomplicated to integrate in small wearable devices. They are ubiquitous in smartwatches and smartphones. Based on our findings vibrotactile devices can potentially be used to evoke a feeling of ownership of external machines.

Electrical stimulation was perceived as uncomfortable due to the particular type of sensation it evokes, which can be a sensation that normally does not occur (or occur rarely) in everyday life. Due to the nature of electrical stimulation, some participants had a low threshold for experiencing the stimulation as unpleasant, hence it was only possible to use a low stimulation level which was experienced as a more local sensation directly below the electrode. Other participants who were more comfortable with the electrical stimulation had higher uncomfortable threshold; hence their stimulation level was perceived as a sensation on a larger area on the finger which better matched the brush stroking path. If the stimulation level could be set to match the sensation of the brush stroking, the sensation would probably be more intuitive and entail higher ratings in the tests. Participants who experienced a tingling sensation along the finger expressed a similar sensation to the brush. In order to experience the electrical stimulation as more comfortable some factors could be changed, e.g., decrease the frequency effectively changing the evoked sensation, or use optimally shaped electrodes.

A limitation of this study is that only a single common control condition was used (one for glabrous skin and one for hairy skin) based on asynchronous stimulation of the rubber hand with a brush. In ideal cases all conditions would also have used an asynchronous control as was used by Tsakiris et al. (2010). However, this was not performed in the current study due to a lack of time. Future studies would be needed to assess if asynchrony for the other conditions would have any effect on the rubber hand illusion.

A3 expressed that he did not feel that the rubber hand belonged to him. He mentioned that after 18 years without a hand, he was well aware of the loss and speculated that he probably could have been more susceptible to the RHI early post-amputation. This is supported by Mayer et al. (2008), where amputees who are aware of their lack of a hand do not consider the prosthesis as their own even if they can see the prosthesis and sense their phantom hand. Ehrsson et al. (2008) mentioned that as years go by without a hand, the amputee’s perceptual system learns to accept the new body image without the hand and becomes less prone to experiencing the RHI.

### 4.1. Proprioceptive drift

The proprioceptive drift was significantly greater with synchronous brush stroking than asynchronous, which is in accordance with the traditional RHI (Botvinick and Cohen, 1998). There was no significant difference in the proprioceptive drift toward the rubber hand between the stimulation modalities.

For some participants, the illusion was strong already after one or two stimulation blocks. This can be seen in the drift pre-test, where the participants almost pointed at the rubber hand’s long finger. This corroborates a prior study (Tsakiris and Haggard, 2005), where a drift towards the rubber hand was shown, even without stimulation. In addition, this drift increased gradually with additional brush stroking (Tsakiris and Haggard, 2005). The gradual drift in our study resulted in a small difference between pre- and post-test and would not show the actual drift towards the rubber hand. Botvinick and Cohen (1998) demonstrated that the accuracy of pointing towards the rubber hand increased in proportion to the reported duration of the illusion. In the present study, these patterns were not predicted since the breaks taken between the stimulation blocks were added with the belief that the illusion would be broken between the stimulation blocks.

A1 had a phantom map, and only 1 year had elapsed since amputation compared to 32 years (A2) and 18 years (A3). Previous studies have shown that amputees with referred sensations reported higher scores on the illusion statements than amputees without, but the illusion scores were not significantly different (Ehrsson et al., 2008). In our study, we observed the same correlation, where A1 experienced higher proprioceptive drift than A2 and A3, see Figure 5. Furthermore, there was no relation between the proprioceptive drift test and the time elapsed since amputation in Ehrsson et al. (2008) study which contradicts our findings that show a decrease in proprioceptive drift with the time elapsed However, in our study we had only three participants with amputations, thus, it is not possible to make strong statements on such a small sample size.

### 4.2. Questionnaire

Previous studies on the RHI have most often applied brush strokes on hairy skin. The volar, i.e., glabrous, parts of the hand and fingers are most important when handling objects and the receptive fields are smaller and thus we think it is important to include these parts when doing experiments where the RHI is induced.

The ownership criterion is arbitrary, but the commonly used is that the illusion statements should score at least one point above the neutral/indifferent rating (Botvinick and Cohen, 1998; Ehrsson, 2004; Kalckert and Henrik Ehrsson, 2012; Kalckert and Ehrsson, 2014), and some only apply this for one of the illusion statements (S3) (Petkova and Ehrsson, 2009). The interpretation of non-responders is assessed differently in previous studies, where some categorize non-responders as those who do not fulfil the ownership criterion, whereas some define responders and non-responders differently. E.g., where the responders had a positive rating (>1) for the ownership statements and non-responders had a negative rating (≤0) (Kalckert and Henrik Ehrsson, 2012) which would leave some participants in the rating scores 0–1, which is not included in either of the categories. If using the cut-off criteria where the mean rating for the illusion statements is ≤0 in this study (including data for both hairy and glabrous skin), there are 5 (18.5%) non-responders for the synchronous brush stroking, 9 (33.3%) for electrical stimulation, 12 (44.4%) for pressure and vibration. Trojan et al. (2018) performed a pre-test to exclude non-responders (20–30%), where a similar criterion was used for the statement S3. Ehrsson (2004) also performed a pre-test to exclude non-responders (28%). The current study showed a higher rate of non-responders compared to previous studies. One possible explanation could be that none of the participants has participated in a previous RHI study, which could contribute to a lower phenomenological control where the participants were unable to generate an experience that would meet the expectancies of the RHI (Roseboom and Lush, 2022). It has been shown that trait phenomenological control can favor RHI ownership statements, suggesting that the questionnaire does not measure ownership but rather measures the ability to generate experiences to meet expectancies (Roseboom and Lush, 2022). Some participants commented” Once I felt an effect of the illusion, I think it increased a bit with time, so maybe the illusion would have been stronger with more time.” This was discussed by Riemer et al. (2019) who analysed and discussed the methodological differences in the RHI and suggested that the assessed RHI onset time (the time when the participant first perceived the feeling of ownership) varied between studies that included and excluded non-responders where the onset time was usually shorter in studies which excluded non-responders. In our study, all participants were included, which would cause skewness in the data compared to studies that exclude non-responders (Riemer et al., 2019). However, the methods in classifying non-responders are based on subjective reports (questionnaire) and not on objective measures (proprioceptive drift), and the literature shows an inconsistency in whether the proprioceptive drift correlates with the ownership ratings (Riemer et al., 2019). Hence, individuals that are classified as non-responders with one measure could classify as responders with another measure (Riemer et al., 2019).

The illusion statement was ranked highest for brush stroking, which provide a modality matched sensory feedback where the brush stroking was applied on both the rubber hand and the hidden hand. Interestingly, there was only a significant difference between brush stroke and pressure and not between brush stroke and electrical stimulation and vibration. Electrical stimulation and vibration had a higher mean rating for the illusion statements than pressure, suggesting the two former modalities matched spatially with the brush stroking on the rubber hand. Moreover, both electrotactile and vibrotactile stimulation deliver dynamic stimuli, which hypothetically provide more activation (firing) of the hand’s mechanoreceptors. Some participants, who had a higher stimulation level for the electrical stimulation, were able to feel a tingling sensation extending along the finger. This imitates how a brush stoke feels to some extent. The vibrotactile stimulation elicits waves that probable propagates across the skin, leaving a sensation on a more extensive area of the finger. Stimulation using pressure induced a weaker RHI than the other stimuli. A possible explanation for this is that pressure is the most spatially mismatched stimulus since the sensation is discrete and more defined than the other stimulation modalities, which gives a sensation in larger areas and is less defined. This would cause a more significant visuotactile conflict in relation to the brush stroke.

### 4.3. Pleasantness

There was no significant difference in pleasantness rating for hairy skin versus glabrous skin. This is somewhat unexpected since prior studies have suggested that the pleasant touch is mediated *via* C-tactile afferents, which are most abundant in hairy skin and that stimulation in the range of 1–10 cm/s is perceived as most pleasant (Löken et al., 2009). However, the current results corroborates some prior studies showing that the pleasantness rating for brush stroking on the palm (glabrous skin) was not significantly different from the arm (hairy skin) if the brush stroking was alternating between the skin types (Löken et al., 2011). For future studies, the pleasantness could be examined by applying stimulation on either glabrous or hairy skin for all types of stimuli, adding a short break, and finishing with the other. This method could show significantly higher rating when stimulating on the hairy skin compared to glabrous skin since the alternation effect would not apply in this case (Löken et al., 2011).

Brush had a significantly higher pleasantness rating than the other stimuli on both hairy and glabrous skin, where stimulation on hairy skin was rated slightly higher than on glabrous skin. This could be explained by brushstrokes giving a light and slow-moving touch, which is effective for activating C-tactile afferents (Nordin, 1990). Stimulation using vibration was ranked as the second most pleasant. Huisman et al. (2016) showed that using multiple vibration motors created a haptic perceptual illusion to spatially match a brush stroke’s path and gave a similar subjective pleasantness rating as actual brushstrokes. Even though vibration does not activate C-tactile afferents, Huisman and colleagues showed that the pleasantness rating followed a U-shape pattern similar to brushstrokes. The current study only used one vibration motor. However, the perceived sensation of the vibration motor covered a large part of the finger, which can be seen as spatially matched to the brush stroke path. Electrical stimulation was the least pleasant, which could be explained by the fact that the majority of the participants were novices to this type of stimulus. Furthermore, electrical stimulation is not a familiar sensation that is encountered in daily life, and thus some may experience it as an uncomfortable and unusual feeling which likely affected the pleasantness rating.

It has been suggested that C-tactile afferents act as a selector to distinguish velocities related to pleasantness during social touch (Morrison et al., 2010). Furthermore, the discriminative processing in the primary (S1) and secondary (S2) somatosensory cortex could influence the tactile processing in the posterior insula (Olausson et al., 2010). In the same way, the affective coding in the insular cortex could modulate responses in S1 and S2. Posterior insula might also be activated during observational touch (Morrison et al., 2011), seeing someone else being stroked by a brush. Based on this fact, seeing the rubber hand receive brushstrokes might affect the pleasantness rating on the other types of stimuli. With this in mind, future studies should investigate the pleasantness rating for the same stimulation modalities, excluding the visual input from the rubber hand to test the tactile feedback solely.

## 5. Conclusion

In this study, we evaluated how different modalities of tactile stimuli (mechanotactile, electrotactile, and vibrotactile), can induce the RHI in able-bodied persons and in forearm amputees. We showed that all stimuli elicit body ownership of a rubber hand to some extent. The RHI became more vivid with tactile stimuli that imitate the stimulation given to the rubber hand. In this case, electrotactile stimulation and vibrotactile could more closely imitate the brush stroking spatially compared to mechanotactile stimulation. A slight drift towards the rubber hand could be seen after completing more stimulation blocks (time spent in one session), which gave unreliable results in the proprioceptive drift test and made it difficult to interpret whether the RHI occurred or not. In contrast to previous studies, the RHI was induced to the same extent if the stimulation was applied on hairy or the glabrous skin. However, future studies are needed to assess if the pleasantness rating depends on the order of the other stimulus types.

## Data availability statement

The raw data supporting the conclusions of this article will be made available by the authors, without undue reservation.

## Ethics statement

The studies involving humans were approved by Swedish Ethical Review Authority (DNR 2021–03630). The studies were conducted in accordance with the local legislation and institutional requirements. The participants provided their written informed consent to participate in this study.

## Author contributions

PS performed the experiments, analysed the results, created the figures, and drafted the manuscript. CA analysed the results and created the figures. NM, UW, AB, and CA provided critical comments and finalized the manuscript for publication. PS, NM, UW, AB, and CA contributed to the design of the study. All authors contributed to the article and approved the submitted version.

## Funding

This work was supported by the Promobilia Foundation, Stiftelsen för bistånd åt rörelsehindrade i Skåne and the Swedish Research Council (DNR 2019–05601).

## Conflict of interest

The authors declare that the research was conducted in the absence of any commercial or financial relationships that could be construed as a potential conflict of interest.

## Publisher’s note

All claims expressed in this article are solely those of the authors and do not necessarily represent those of their affiliated organizations, or those of the publisher, the editors and the reviewers. Any product that may be evaluated in this article, or claim that may be made by its manufacturer, is not guaranteed or endorsed by the publisher.
