# Supplementary figures and images for: The rubber hand illusion evaluated using different stimulation modalities
Source: Front Neurosci. 2023 Sep 14;17:1237053. doi: 10.3389/fnins.2023.1237053 (PMC10536259; doi:10.3389/fnins.2023.1237053)

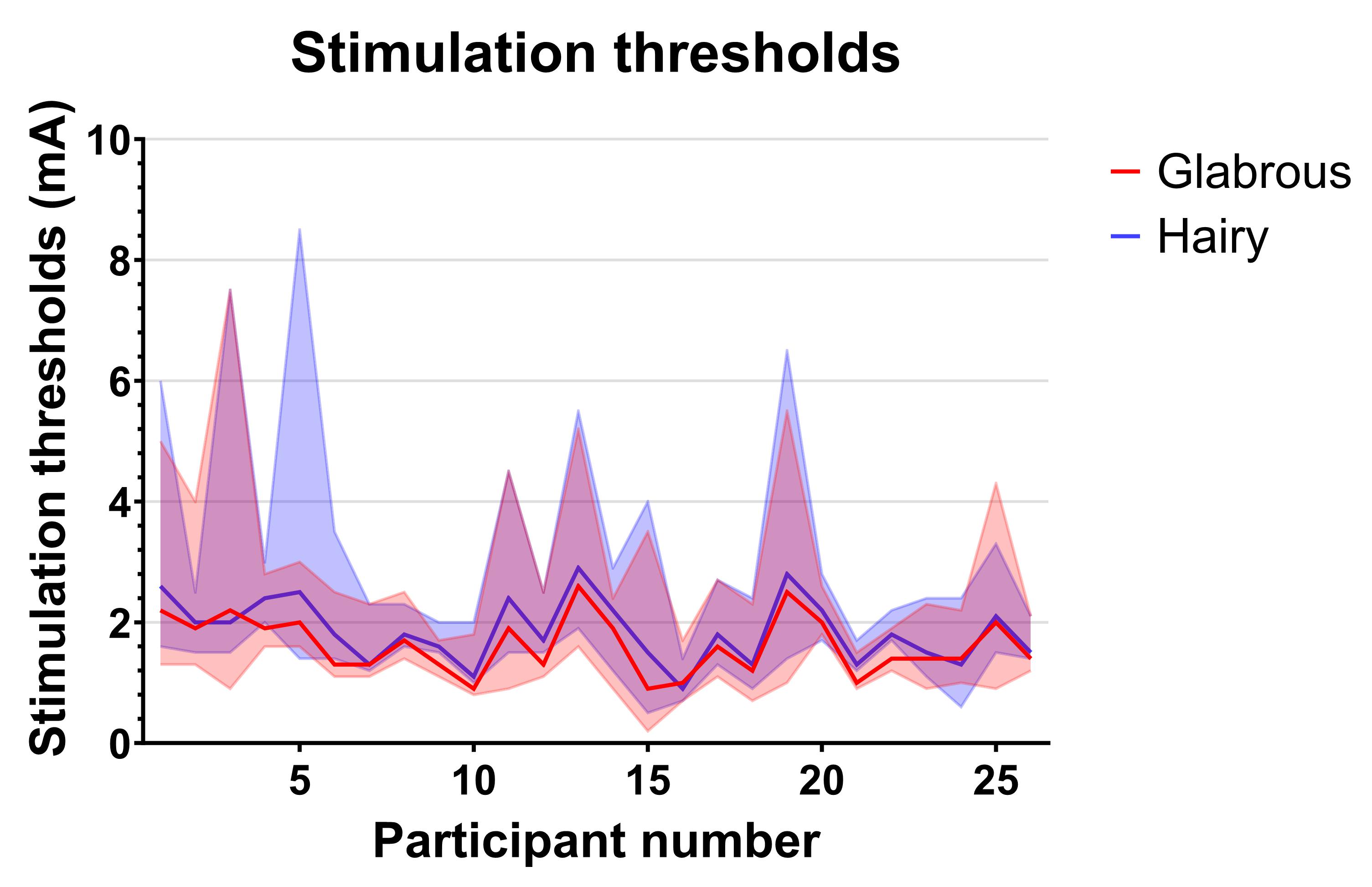

Supplement: Supplementary file 1 [file Image_1.JPEG]
